# Treatment patterns and prognosis of patients with inoperable stage III NSCLC after completion of concurrent chemoradiotherapy ± immune checkpoint inhibition: a decade-long single-center historical analysis

**DOI:** 10.1007/s00432-022-04174-z

**Published:** 2022-08-02

**Authors:** Benedikt Flörsch, Julian Taugner, Lukas Käsmann, Saskia Kenndoff, Julian Guggenberger, Amanda Tufman, Niels Reinmuth, Thomas Duell, Claus Belka, Chukwuka Eze, Farkhad Manapov

**Affiliations:** 1 Department of Radiation Oncology, University Hospital, LMU Munich, Marchioninistraße 15, 81377 Munich, Germany; 2 Member of the German Center for Lung Research (DZL), Comprehensive Pneumology Center Munich (CPC-M), Munich, Germany; 3 German Cancer Consortium (DKTK), Partner Site Munich, Munich, Germany; 4 Division of Respiratory Medicine and Thoracic Oncology, Department of Internal Medicine V, Thoracic Oncology Centre Munich, LMU Munich, Munich, Germany; 5 Asklepios Kliniken GmbH, Asklepios Fachkliniken Munich, Gauting, Germany

**Keywords:** Lung cancer, Thoracic radiotherapy, Outcome, Planning target volume, Immune checkpoint inhibition

## Abstract

**Purpose:**

To investigate the impact of treatment time and patterns in inoperable stage III non-small cell lung cancer (NSCLC) following concurrent chemoradiotherapy (cCRT) ± immune checkpoint inhibitors (ICIs).

**Methods:**

Patients were stratified by treatment year: A (2011–2014), B (2015–2017) and C (2018–2020). Tumor- and treatment-related characteristics regarding locoregional recurrence-free survival (LRRFS), progression-free survival (PFS) and overall survival (OS) were investigated.

**Results:**

One hundred and thirty-six consecutive patients were analyzed. All patients completed thoracic radiotherapy (TRT) to a total dose ≥ 60.0 Gy; 36 (26%) patients received ICI. Median PFS in subgroups A, B and C was 8.0, 8.2 and 26.3 months (*p* = 0.007). Median OS was 19.9 months, 23.4 months and not reached (NR), respectively. In group C, median LRRFS and PFS were 27.2 vs. NR; and 14.2 vs. 26.3 months in patients treated with and without ICI. On multivariate analysis planning target volume (PTV) ≥ 700 cc was a negative prognosticator of LRRFS (HR 2.194; *p* = 0.001), PFS (HR 1.522; *p* = 0.042) and OS (HR 2.883; *p* = 0.001); ICI was a predictor of LRRFS (HR 0.497; *p* = 0.062), PFS (HR 0.571; *p* = 0.071) and OS (HR 0.447; *p* = 0.1). In the non-ICI cohort, multivariate analyses revealed PTV ≥ 700 cc (*p* = 0.047) and a maximum standardized uptake value (SUV_max_) ≥ 13.75 (*p* = 0.012) were predictors of PFS; PTV ≥ 700 cc (*p* = 0.017), SUV_max_ ≥ 13.75 (*p* = 0.002) and a total lung V20 ≥ 30% (V20 ≥ 30) (*p* < 0.05) were predictors of OS.

**Conclusions:**

Patients treated after 2018 had improved survival regardless of ICI use. Implementation of ICI resulted in further significant increase of all tested survival endpoints. PTV ≥ 700 cc and ICI were only prognosticators for LRRFS, PFS and OS in the analyzed cohort.

## Introduction

Lung cancer is the most common cause of cancer-related deaths worldwide (Siegel et al. 2021). Regarding inoperable stage III non-small cell lung cancer (NSCLC), prognoses differ widely in patients with inoperable stage III NSCLC depending on multiple patient- and treatment-related factors. Historically, outcome has been poor, with 10–30% of patients with inoperable stage III NSCLC surviving after 5 years following multimodal therapy (Taugner et al. 2019, 2020; Käsmann et al. 2019). Chemoradiotherapy (CRT) had been the standard treatment for over three decades (Group BMJP 1995). Guidelines recommend concurrent over sequential CRT, since it has been shown to deliver more favorable loco-regional control and overall survival (OS) (Postmus et al. 2017; Remon et al. 2021). Since 2017, treatment recommendations for patients with inoperable stage III NSCLC combine concurrent chemoradiotherapy with consecutive maintenance treatment with programmed cell death ligand 1 (PD-L1) inhibitor durvalumab.

The incorporation of immune-checkpoint inhibition (ICI) in multimodal treatment strategies led to significant improvement in patient outcomes. The PD-L1 inhibitor durvalumab led to a paradigm shift following the unprecedented results of the PACIFIC trial with a median progression-free survival (PFS) of 16.9 months and a median OS of 47.5 months (Antonia et al. 2017; Spigel et al. 2022). Our understanding regarding the impact of other innovations in the diagnostic and treatment such as the introduction of volumetric modulated arc therapy (VMAT) and strict treatment planning constraints under real-world conditions remains limited. Therefore, we identified tumor- and treatment-related variables and performed a retrospective analysis of outcomes in patients that finished concurrent CRT (cCRT) for inoperable stage III NSCLC at a single tertiary cancer center over the last decade. Furthermore, we divided our patient cohort into three subgroups depending on the time of treatment and analyzed outcomes based on this grouping. The goal of this study was to further the understanding of treatment-associated parameters and to provide new impulses for scientific approaches and treatment patterns.

## Methods

**Fig. 1:**
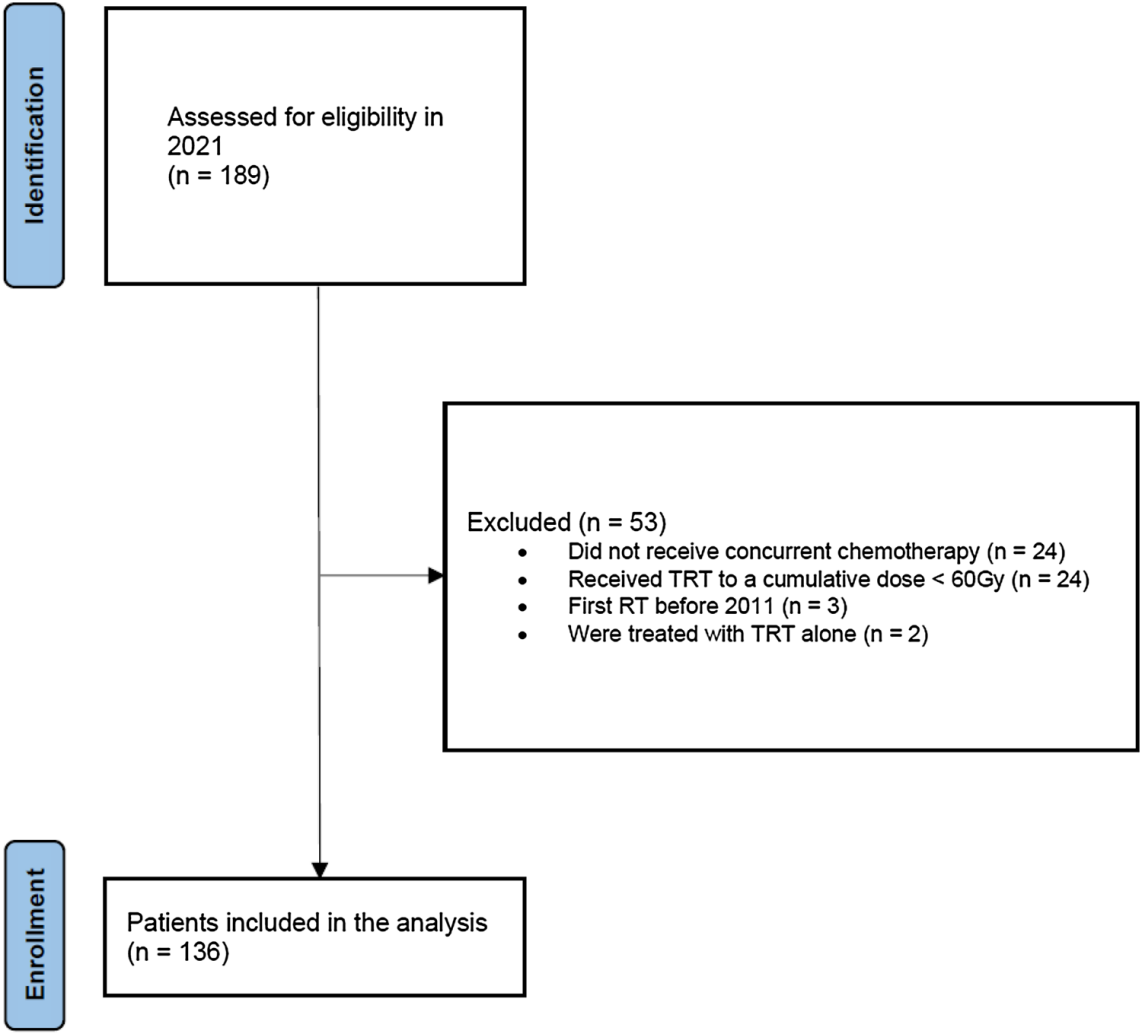
STROBE flowchart for patient accrual

Records of 189 consecutive patients with stage III NSCLC were screened. A total of 136 (71.9%) from 189 consecutive patients treated between February 2011 and November 2020 were enrolled in the study. Eligible patients were diagnosed with inoperable stage IIIA-C (UICC 8th edition) NSCLC and received cCRT ± ICI as part of a multimodal approach. ICI consisted of either consolidation administration of durvalumab according to the PACIFIC trial or concurrent and sequential administration of nivolumab as part of the ETOP 6–14 NICOLAS phase II study (Peters et al. 2019, 2021). All patients provided informed consent. The local ethics committee approved the retrospective collection and analysis of patient data (reference number: 17-230). The detailed flowchart is presented in Fig. 1.

All patients received cCRT consisting of two cycles of platinum-based chemotherapy (cisplatin/carboplatin combined with vinorelbine/pemetrexed) and conventionally fractionated thoracic radiotherapy (TRT) to a total dose of ≥ 60 Gy. Analysis of all parameters was performed for the entire cohort and additionally for patients that had not received ICI (non-ICI cohort). Patients were stratified by year of initial TRT and divided into three subgroups: subgroup A (2011–2014), subgroup B (2015–2017) and subgroup C (2018–2020). We analyzed treatment- and patient-related characteristics: age ≥ 65, application of VMAT, use of ICI, tumor histology, planning target volume (PTV) ≥ 700 cc, maximum standardized uptake value (SUV_max_) ≥ 13.75 (cutoff defined by median dichotomization of all maximum standard uptake values), total lung V20 ≥ 30% (V20 ≥ 30) and smoking history ≥ 20 pack years in regard to locoregional recurrence-free survival (LRRFS), PFS and overall survival (OS). All survival parameters were calculated from the last day of TRT.

In 133 (97.8%) patients, baseline staging was performed using positron emission tomography (PET)-computed tomography (CT). Cranial contrast-enhanced MRI was performed in 88 (64.7%) patients before starting multimodal treatment, all other patients received cranial contrast-enhanced CT. Pulmonary function testing and routine blood work were performed in all patients. All patients were discussed at the multidisciplinary tumor board and deemed eligible for cCRT. Eligible patients were required to have an Eastern Cooperative Oncology Group (ECOG) performance status (PS) of 0–1 and adequate lung function: diffusing capacity of lung for carbon monoxide corrected for hemoglobin (DLCOc) ≥ 40%, forced expiratory volume in 1 s (FEV1) ≥ 1 L. Treatment plans for TRT were based on conventional planning-CT and PET-CT. These scans were acquired with patients in the corresponding treatment position. Patients were treated in the supine position with their arms positioned overhead in a dedicated positioning and immobilization device-WingSTEP™ (Innovative Technologie Voelp, Innsbruck, Austria). Target volumes were defined according to an in-house protocol in close accordance with the later published guidelines of the European Society for Radiotherapy and Oncology-Advisory Committee on Radiation Oncology Practice (ESTRO-ACROP) (Nestle et al. 2018).

Until the introduction of intensity-modulated radiotherapy (IMRT) for lung treatments, TRT consisted of 50 Gy in 2 Gy single-dose fractions, followed by a sequential 16 Gy boost. After the full implementation of IMRT, TRT-regimens consisted of 30 fractions with simultaneous integrated boost (SIB) of 2.0/2.12 Gy to the gross tumor volume (GTV) lymph node (LN)/GTV primary tumor (PT) to a total dose of 60.0/63.6 Gy. TRT was delivered on a linear accelerator (LINAC) with megavoltage capability, using either VMAT, 3D-CRT or step-and-shoot IMRT. Image guidance was performed at least twice a week on a cone-beam CT.

ICI maintenance treatment was administered for a duration of up to 12 months and/or until disease progression or evidence of unacceptable toxicity. Durvalumab was given at a dose of 10 mg/kg of body weight every two weeks; nivolumab was administered concurrently at a dose of 360 mg, every third week and after CRT at a dose of 480 mg, monthly up to 1 year.

Diagnostic measures like routine blood work, lung function testing, clinical examination and imaging with CT or PET-CT scans were performed every 3 months in the first 2 follow-up years after treatment, every 6 months in the following 2 years and annually thereafter. Additional imaging, e.g. contrast-enhanced magnetic resonance imaging (MRI) and bone-scintigraphy, was performed if deemed necessary.

Tumor progression was assessed according to RECIST 1.1. CT, PET-CT or MRI scans were used to document local relapse (LR), while pathological confirmation was not obligatory. LR was defined as recurrence in the ipsilateral lung or mediastinum. Median follow-up was defined as the time from the end of TRT to loss or end of follow-up in patients that had not died up to that point. LRRFS was defined as the time from the end of TRT until either LR or death. Similarly, PFS was defined as the time from the end of TRT to locoregional/systemic progression or death, while OS was defined as the time to death from any cause or last follow-up. Volumetric parameters were derived from the radiation treatment plans. The impact of each parameter on LRRFS, PFS and OS was analyzed by means of Kaplan–Meier analysis using the log-rank test. Multivariable Cox-regression analysis was performed with all parameters, which had shown to be significant (*p* < 0.05) in univariate analysis. All analyses including univariate and multivariate analysis were performed using SPSS version 26 (IBM; Armonk, New York, USA).

## Results

Between 2011 and 2020, a total of 136 consecutive patients with inoperable stage IIIA-C (UICC 8th edition) NSCLC were analyzed. A summary of patient- and tumor characteristics is shown in Table 1.

**Table 1:** Patient characteristics (entire cohort)

	Number of patients (%)
Age	
< 65 years	58 (43)
≥ 65 years	78 (57)
Gender	
Female	43 (32)
Male	93 (68)
UICC stage	
IIIA	41 (30)
IIIB	52 (38)
IIIC	43 (32)
T category	
1–2	33 (24)
3–4	100 (74)
N/A	3 (2)
N category	
0–1	26 (19)
2–3	110 (81)
Histology	
Squamous cell carcinoma	56 (41)
Adenocarcinoma	69 (51)
Not otherwise specified (NOS)	11 (8)
Over 20 pack-years	
Yes	92 (68)
No	21 (15)
Not reported	23 (17)
Year of TRT	
Subgroup A: 2011–2014	37 (27)
Subgroup B: 2015–2017	49 (36)
Subgroup C: 2018–2020	50 (37)
Radiation technique	
VMAT	82 (60)
Step-and-shoot IMRT	19 (14)
3D-conformal RT	35 (26)
Planning target volume	
< 700 cc	68 (50)
≥ 700 cc	68 (50)
Median SUV_max_	
≥ 13.75	59 (43)
< 13.75	59 (43)
N/A	18 (14)
Induction chemotherapy	
Yes	60 (44)
No	76 (56)
Immunotherapy	
Yes	36 (26)
No	100 (74)

### Patient characteristics

Subgroup A consisted of 37 (27.2%) patients, who received TRT between 2011 and 2014, subgroup B consisted of 49 (36.0%) patients, that were treated between 2015 and 2017 and subgroup C consisted of 50 (36.8%) patients with treatment between 2018 and 2020. The median age was 66.9 (range 33.6–82.5) years with 78 (57%) patients older than 65 years. Forty-three (32%) were female and 93 (68%) were male. Fifty-six (41%) patients had squamous cell carcinoma (SCC), 69 (51%) had adenocarcinoma (AC) and in 11 (8%) patients the tumor was classified as not otherwise specified (NOS); 92 (68%) patients were current or former smokers with more than 20 pack-years, 21 (15%) had less than 20 pack-years and for 23 (17%) patients the smoking status could not be discerned. All patients completed conventionally fractionated radiotherapy to a total dose ≥ 60.0 Gy. Of these, 82 (60%) were treated with VMAT, while 35 (26%) received 3D-CRT and 19 (14%) patients were treated with step-and-shoot IMRT. The median PTV was 700.5 cc (range 172.5–2293.2). Sixty-eight (50%) patients had a PTV ≥ 700 cc. SUV_max_ was available for 118 (87%) patients and was ≥ 13.75 in 59 (43%) cases. All patients finished cCRT, with 60 (44%) receiving induction chemotherapy. Thirty-six (26%) patients received either durvalumab or nivolumab. In the entire cohort, median follow-up was 35.7 (range 0.9–111.9) months, median LRRFS, 12- and 18-month LRRFS rates were 23.2 (95% CI 14.9–31.4), 58% and 45%, respectively. Median PFS, 12- and 18-month PFS rates were 10.1 months (95% CI 7.4–12.8), 38% and 27%, respectively. Median OS, 12- and 18-month OS rates were 27.4 months (95% CI 16.0–38.7), 70% and 54%, respectively (Table 2).

**Table 2:** Survival parameters

	Number of patients (%)
Median follow-up (months)	35.7
LRRFS entire cohort	
Median (months)	23.2
12-month	79 (58)
18-month	61 (45)
Median LRRFS (months) by subgroup	
A (2011–2014)	14.1
B (2015–2017)	16.9
C (2018–2020)	NR
PFS	
Median (months)	10.1
12-month	52 (38)
18-month	37 (27)
Median PFS (months) by subgroup	
A (2011–2014)	8.0
B (2015–2017)	8.2
C (2018–2020)	26.3
OS	
Median (months)	27.4
12-month	95 (70)
18-month	74 (54)
Median OS (months) by subgroup	
A (2011–2014)	19.9
B (2015–2017)	23.4
C (2018–2020)	NR

For detailed results of the uni- and multivariate analyses, refer to Tables 3 and 4.

**Table 3:** Univariate analysis

Patient cohort	Parameter	Univariate analysis (*p* value)		
		LRRFS	PFS	OS
Entire cohort (*n* = 136)	Treatment groups	0.007	0.007	0.001
	ICI	0.004	0.002	0.001
	Age ≥ 65	0.151	0.609	0.016
	VMAT	0.012	0.001	0.001
	Histology	0.057	0.425	0.196
	PTV ≥ 700	0.000	0.011	0.001
	SUV_max_ ≥ 13.75	0.161	0.076	0.163
	V20 ≥ 30	0.003	0.030	0.002
	20 + PY	0.566	0.487	0.042
Non-ICI (*n* = 100)	Treatment groups	0.323	0.095	0.083
	Age ≥ 65	0.215	0.442	0.057
	VMAT	0.268	0.051	0.121
	Histology	0.111	0.504	0.393
	PTV ≥ 700	0.001	0.042	0.007
	SUV_max_ ≥ 13.75	0.015	0.019	0.027
	V20 ≥ 30	0.016	0.060	0.002
	20 + PY	0.378	0.369	0.097
Subgroup C (2018–2020)	ICI	0.226	0.629	0.583

**Table 4:** Multivariate analysis

Patient cohort	Parameter	Multivariate analysis					
		LRRFS HR (95% CI)	*p* value	PFS (HR (95% CI)	*p* value	OS HR (95% CI)	*p* value
Entire cohort (*n* = 136)	Treatment groups	0.851 (0.516–1.404)	0.528	1.123 (0.711–1.776)	0.619	0.820 (0.439–1.534)	0.535
	ICI	0.497 (0.239–1.035)	0.062	0.571 (0.311–1.050)	0.071	0.447 (0.168–1.191)	0.100
	Age ≥ 65 years					2.228 (1.197–4.146)	0.011
	VMAT	1.125 (0.515–2.459)	0.767	0.599 (0.283–1.271)	0.182	1.065 (0.421–2.695)	0.894
	PTV ≥ 700 cc	2.194 (1.399–3.441)	0.001	1.522 (1.016–2.279)	0.042	2.883 (1.645–5.050)	0.001
	V20 ≥ 30	1.755 (1.024–3.009)	0.410	1.422 (0.847–2.387)	0.183	1.497 (0.727–3.080)	0.274
	20 + PY					2.546 (1.061–6.111)	0.036
Non-ICI (*n* = 100)	PTV ≥ 700 cc	2.065 (1.215–3.509)	0.007	1.630 (1.007–2.639)	0.047	1.958 (1.126–3.404)	0.017
	SUV_max_ ≥ 13.75	2.252 (1.330–3.813)	0.003	1.859 (1.144–3.021)	0.012	2.405 (1.366–4.234)	0.002
	V20 ≥ 30	2.248 (1.243–4.066)	0.007			3.357 (1.780–6.330)	0.000

### Univariate analysis of the entire cohort

In the univariate analysis for the entire cohort, stratification into treatment groups A, B and C was significantly associated with established endpoints. Namely, group C had significantly longer LRRFS (*p* = 0.007), PFS (*p* = 0.007) and OS (*p* = 0.001). Median LRRFS was 14.1 (95% CI 7.7–20.5) months in subgroup A, 16.9 (range 7.9–25.9) months in subgroup B and not reached in subgroup C. Median PFS was 8.0 (95% CI 6.6–9.4), 8.2 (95% CI 2.8–13.6) and 26.3 (95% CI 7.5–45.1) months for groups A, B and C, respectively. Median OS for subgroups A and B was 19.9 (95% CI 10.7–29.1) and 23.4 (95% CI 15.1–31.6) months, whereas it was not reached for C.

In patients that received no ICI median LRRFS was 17.0 (95% CI 9.4–24.5) months vs. not reached for patients that received ICI (*p* = 0.004). Median PFS was 8.1 (95% CI 5.9–10.2) months vs. 22.8 (95% CI 7.8–37.7), respectively (*p* = 0.002). Median OS was 21.1 (95% CI 15.4–26.7) months vs. not reached (*p* = 0.001).

Median LRRFS of patients over 65 was 23.2 (95% CI 14.4–31.9) months vs. 21.2 (95% CI 5.3–37.0) months in patients under the age of 65 (*p* = 0.151). Median PFS was 9.8 (95% CI 7.1–12.5) vs. 11.8 (95% CI 5.4–18.2) months for patients over and under 65 years (*p* = 0.609). However, median OS was 23.4 (95% CI 16.3–30.5) vs. 73.4 (95% CI 0.3–146.5) months (*p* = 0.016) for patients ≥ 65 and < 65, respectively.

We analyzed the use of VMAT in the entire cohort and observed significantly longer survival in patients that received VMAT vs. patients with other forms of TRT. Median LRRFS was 30.3 (95% CI 15.2–45.4) vs. 12.1 (95% CI 4.7–19.4) months (*p* = 0.012). Median PFS was 14.0 (95% CI 3.4–24.6) vs. 7.4 (95% CI 5.1–9.7) months (*p* = 0.001), and median OS was 52.2 (95% CI 9.9–94.5) vs. 19.9 (95% CI 14.2–25.6) months, respectively (*p* = 0.001).

Univariate analysis of tumor histology revealed no significant association between histology and any of the endpoints; however, there was a trend toward longer LRRFS for patients with AC (*p* = 0.057).

PTV ≥ 700 cc was significantly associated with all endpoints. Median LRRFS of patients with PTV ≥ 700 cc vs. patients with PTV < 700 cc was 11.4 (95% CI 8.9–13.9) vs. 43.1 (95% CI 22.8–63.5) months (*p* < 0.05). Median PFS was 6.9 (95% CI 4.8–9.1) vs. 15.4 (95% CI 9.0–21.7) months (*p* = 0.011), and median OS was 16.3 (95% CI 10.8–21.8) vs. 52.2 (95% CI 27.9–76.6) months (*p* = 0.001).

We found no significant association between SUV_max_ ≥ 13.75 and the established endpoints.

V20 ≥ 30 was 6.1% vs. 31.5% in patients treated with vs. without VMAT. Similarly, 11.8% vs. 20.6% of patients had V20 ≥ 30 with PTV < vs. ≥ 700 cc.

Median LRRFS in the entire cohort was 8.9 (95% CI 2.9–15.0) months for patients with total lung V20 ≥ 30% vs. 27.2 (95% CI 18.1–36.3) months for patients with V20 < 30% (*p* = 0.003). Similarly, median PFS was 5.8 (95% CI 0.0–12.2) vs. 11.0 (95% CI 8.6–13.4) months (*p* = 0.030), and median OS was 12.2 (95% CI 7.5–16.8) months vs. 34.9 (95% CI 16.0–53.8) months (*p* = 0.002).

Median OS was 27.4 (95% CI 13.6–41.1) vs. 77.9 (95% CI 0.4–155.4) months for patients with more vs. patients with less than 20 pack-years (*p* = 0.042).

### Univariate analysis of the non-ICI cohort 

In patients without ICI, stratification by treatment year did not show a significant association with LRRFS (*p* = 0.323); however, we observed a trend for longer PFS (*p* = 0.095) and OS (*p* = 0.083) in subgroup C.

We observed no significant association between age ≥ 65 and LRRFS or PFS amongst patients in the non-ICI cohort, but a trend toward longer OS for patients under 65 (*p* = 0.057).

There was no significant association of VMAT with LRRFS and OS amongst patients in the non-ICI cohort but a trend toward longer PFS (*p* = 0.051).

We observed no significant association between tumor histology and established endpoints in patients without ICI.

In subgroup analysis of patients without ICI, median LRRFS was 9.5 (95% CI 7.2–11.9) months for patients with PTV ≥ 700 cc and 37.8 (95% CI 16.0–59.5) months for patients with PTV < 700 cc (*p* = 0.001). Median PFS was 6.2 (95% CI 3.5–8.9) and 11.2 (95% CI 6.9–15.6) months, respectively (*p* = 0.042). Median OS was 14.7 (95% CI 10.0–19.5) months and 43.1 (95% CI 15.9–70.3) months, respectively (*p* = 0.007).

SUV_max_ ≥ 13.75 was significantly associated with all three endpoints in the non-ICI cohort. Median LRRFS amounted to 8.4 (95% CI 6.0–10.7) months for patients with SUV_max_ ≥ 13.75 vs. 25.1 (95% CI 14.1–36.0) months, for patients with SUV_max_ < 13.75 (*p* = 0.015). Median PFS was 5.4 (95% CI 3.5–7.2) vs. 14.1 (95% CI 5.6–22.8) months (*p* = 0.019), and median OS was 15.4 (95% CI 9.9–20.9) months and 31.2 (95% CI 9.4–53.1) months, respectively (*p* = 0.027).

**Fig. 2:**
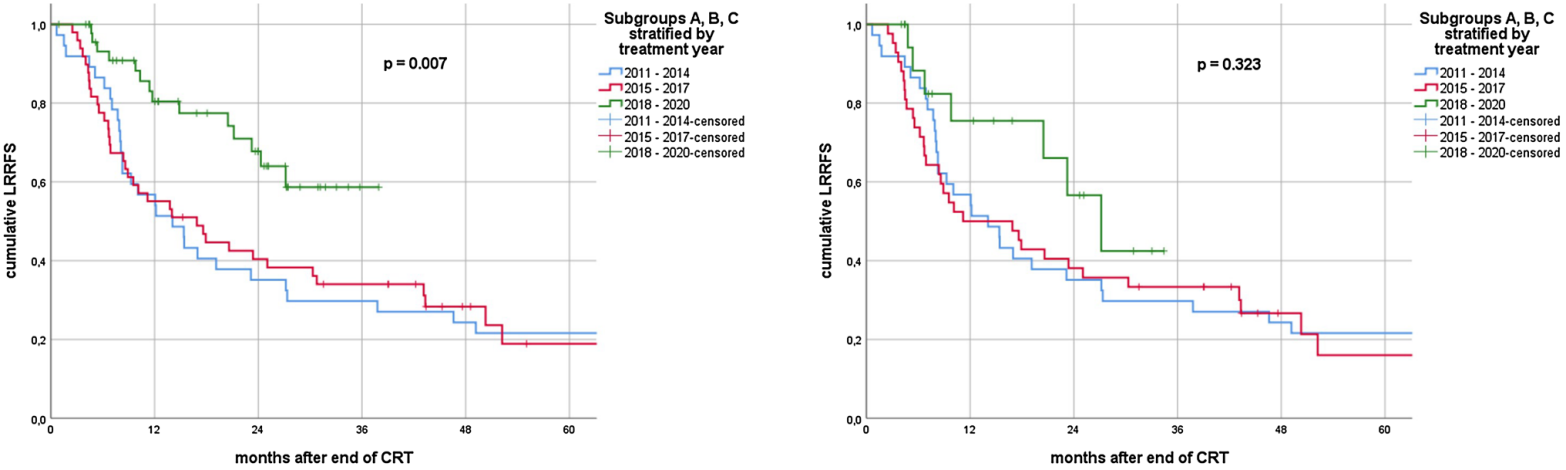
Kaplan–Meier curves of loco-regional recurrence-free survival (LRRFS) for all patients (left) vs. patients without ICI (right) stratified by treatment year groups

**Fig. 3:**
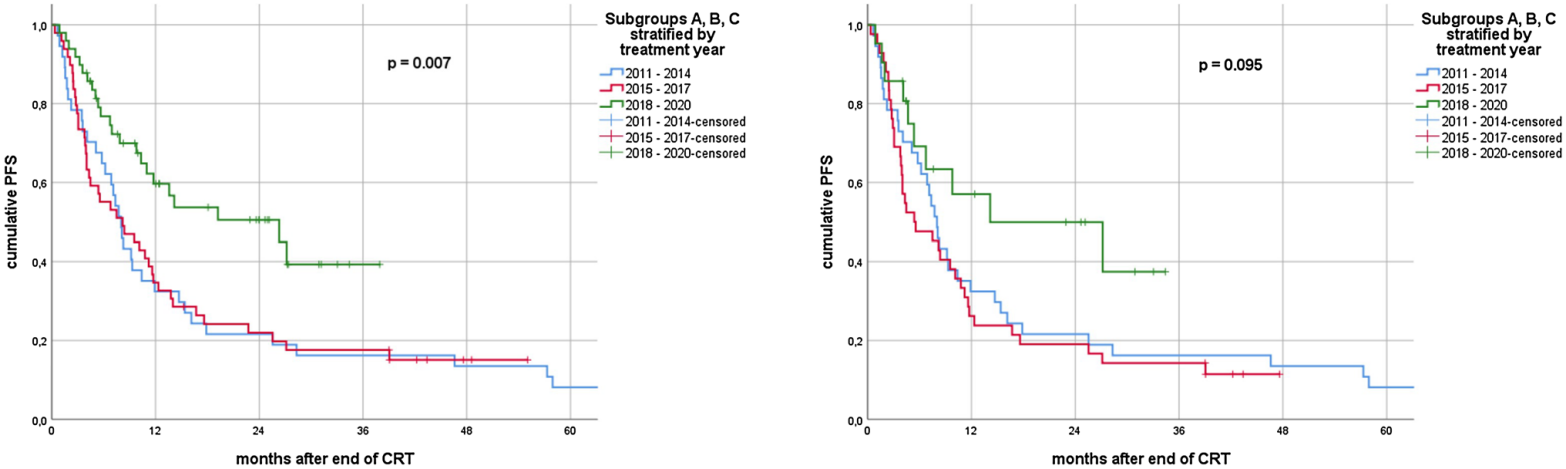
Kaplan–Meier curves of progression-free survival (PFS) for all patients (left) vs. patients without ICI (right) stratified by treatment year groups

**Fig. 4:**
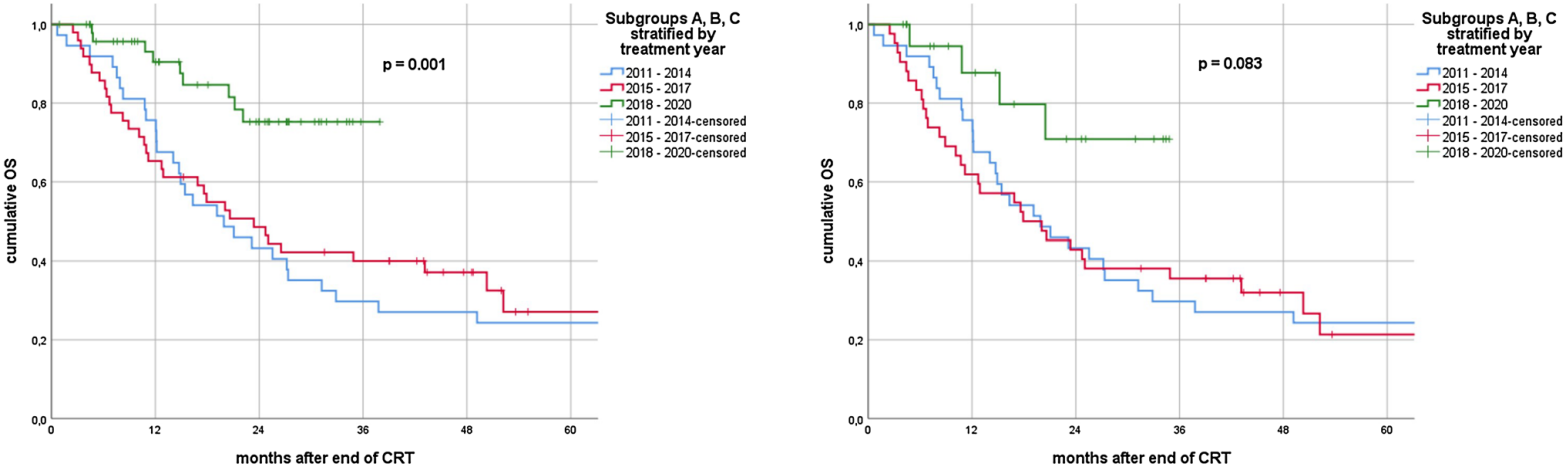
Kaplan–Meier curves of overall survival (OS) for all patients (left) vs. patients without ICI (right) stratified by treatment year groups

Median LRRFS was 8.3 (95% CI 7.0–9.5) vs. 20.5 (95% CI 13.2–27.8) months for patients with V20 ≥ 30 and V20 < 30, respectively (*p* = 0.016). Median OS amounted to 12.1 (95% CI 7.6–16.7) months and 25.6 (95% CI 14.6–36.5) months (*p* = 0.002). Furthermore, we observed a trend toward shorter median PFS for patients with V20 ≥ 30: 5.6 (95% CI 1.1–10.0) vs. 9.2 (95% CI 6.6–11.7) months (*p* = 0.060). (Figs. 2, 3, 4).

### Univariate analysis of patient-subgroup C

In subgroup C, median LRRFS was 27.2 (95% CI 18.4–35.9) months for patients without ICI, while it was not reached for patients that had received ICI (*p* = 0.226). Median PFS for patients with no ICI was 14.2 (95% CI 0.0–35.4) months and 26.3 (95% CI 6.7–45.9) months for patients with ICI (*p* = 0.629). Median OS was not reached for patients of subgroup C, regardless of ICI use (*p* = 0.583).

### Multivariate analysis

Multivariate analysis for the entire cohort included stratification by treatment year, ICI, age ≥ 65 years, application of VMAT, PTV ≥ 700 cc, V20 ≥ 30 and 20 + PY.

On multivariate analysis, PTV ≥ 700 cc remained significantly associated with LRRFS [HR 2.194 (95% CI 1.399–3.441, *p* = 0.001)] and PFS [HR 1.522 (95% CI 1.016–2.279, *p* = 0.042)]. For OS, age ≥ 65 years [HR 2.228 (95% CI 1.197–4.146, *p* = 0.011)], PTV ≥ 700 cc [HR 2.883 (95% CI 1.645–5.050, *p* = 0.001)] and 20 + PY [HR 2.546 (95% CI 1.061–6.111, *p* = 0.036)] remained significant prognosticators. Furthermore, there was a clear trend toward longer LRRFS [HR 0.497 (95% CI 0.239–1.035, *p* = 0.062)], PFS [HR 0.571 (95% CI 0.311–1.050, *p* = 0.071)] and OS [HR 0.447 (95% CI 0.168 1.191, *p* = 0.100)] in patients treated with ICI.

In patients without ICI, PTV ≥ 700 cc was also associated with LRRFS [HR 2.065 (95% CI 1.215–3.509, *p* = 0.007)], PFS [HR 1.630 (95% CI 1.007–2.639, *p* = 0.047)] and OS [HR 1.958 (95% CI 1.126–3.404, *p* = 0.017)]. Similarly, SUV_max_ ≥ 13.75 remained a prognosticator of LRRFS [HR 2.252 (95% CI 1.330–3.813, *p* = 0.003)], PFS [HR 1.859 (95% CI 1.144–3.021, *p* = 0.012)] and OS [HR 2.405 (95% CI 1.366–4.234, *p* = 0.002)]. V20 ≥ 30 was significantly associated with LRRFS [HR 2.248 (95% CI 1.243–4.066, *p* = 0.007)] and OS [HR 3.357 (95% CI 1.780–6.330, *p* < 0.05)].

## Discussion

The aim of the present study was to compare oncological outcome in inoperable stage III NSCLC patients after cCRT ± ICI depending on the treatment time and pattern. Additionally, impact of different tumor- and treatment-related factors on patient prognosis was analyzed separately in the entire cohort and in the cohort treated without ICI (non-ICI).

Consecutive patients that completed cCRT ± ICI at a single tertiary cancer center from 2011 to 2020 were evaluated. Initial staging and treatment planning were performed according to an in-house protocol in close accordance with the published ESTRO-ACROP guidelines (Nestle et al. 2018). The majority of patients (97.8%) underwent 18F-FDG PET/CT in the treatment position prior to multimodal therapy, which was used for both exact definition of UICC stage and delineation of target volumes. In the case of induction chemotherapy, imaging before and after was taken into consideration (Nestle et al. 2021). All patients had contrast-enhanced cranial MRI or CT as a part of initial tumor staging.

The present study revealed that multimodal treatment applied from 2018 to 2020 was associated with improved long-term patient outcome independently of the use of ICI when compared to treatment prior to 2018. Concretely, PFS and OS were continuously better in subgroup C independent of the use of ICI. However, the addition of ICI has further potentiated this positive effect. Thirty-six patients who completed tri-modal therapy after 2018 achieved a median PFS of 26.3 compared to 14.2 months in patients treated without ICI.

Generally, these findings are in line with an analysis by Hansen et al. which demonstrated a steady improvement in survival for patients with unresectable stage III NSCLC treated between 2000 and 2013 (Hansen et al. 2020). A feature of the present study is the continuous improvements following the implementation of ICI regarding all tested endpoints (LRRFS, PFS and OS).

Ground-breaking results of the phase III PACIFIC trial have led to the prompt establishment of cCRT followed by maintenance treatment with PD-L1 inhibitor durvalumab as a new tri-modal standard of care for unresectable stage III NSCLC. Maintenance treatment with durvalumab in patients without PD after cCRT was associated with an unprecedented increase in local and distant tumor control as well as long-term outcome. Subsequently, several real-world data confirmed the efficacy and safety of this new tri-modal approach. Notwithstanding the crucial role of durvalumab, there is a need to evaluate other important tumor- and treatment-related factors within this new treatment paradigm.

While the present findings should be approached with caution, given the limited number of patients and the retrospective design, we found that multiple factors such as year of treatment, age ≥ 65 years, PTV ≥ 700 cc, ICI, application of VMAT, V20 ≥ 30 and 20 + PY are associated with various survival endpoints. However, in multivariate analysis of the entire cohort, only PTV ≥ 700 cc and use of ICI were independent predictors of LRRFS, PFS and OS.

In the Radiation Therapy Oncology Group 93-11 Phase I–II dose-escalation study, small tumors (< 45 cc) were associated with longer median survival time and superior PFS compared to patients with larger tumors (29.7 vs 13.3 months, *p* < 0.0001 and 15.8 vs 8.3 months, *p* < 0.0001) (Werner-Wasik et al. 2008). A widely recognized trial confirming PTV as an important prognosticator was the RTOG 0617 study (Bradley et al. 2015, 2020). In this open-label randomized phase III study, 419 patients with inoperable stage III NSCLC underwent cCRT between 2007 and 2011. Univariate analysis demonstrated significant impact of both GTV and PTV on risk of death. Furthermore, PTV was a significant prognosticator for OS in the multivariate analysis (Bradley et al. 2015). Further updates of this study confirmed that smaller PTV was associated with significantly better OS (Bradley et al. 2020). A post hoc analysis by Chen et al., regarding radiation treatment technique in RTOG 0617 observed that while survival is similar between patients receiving IMRT vs. 3D-CRT, IMRT was associated with significantly lower rates of severe pneumonitis and cardiac doses. Additionally, Liu et al. further reported that the use of VMAT led to significantly lower dose-volume parameters in normal tissue while achieving similar efficacy compared to IMRT (Chun et al. 2017; Liu et al. 2021).

There are little data concerning the role of PTV in unresectable stage III NSCLC patients treated with tri-modal therapy. We previously demonstrated that the effect of ICI on PFS was weaker in patients with larger tumors. Patients with PTV ≥ 900 cc achieved significantly worse PFS and extracranial distant metastasis-free survival (eDMFS) especially when large PTV was combined with UICC stage IIIC disease (Taugner et al. 2021). The PACIFIC trial does not provide comprehensive information about treated tumor volumes and the impact of PTV on patient outcome (Antonia et al. 2018, 2017; Gray et al. 2020; Hui et al. 2019; Faivre-Finn et al. 2021). Additionally, the non-randomized phase II trials LUN 14-179 and NICOLAS have described a significantly worse OS in patients with UICC stage IIIA vs IIIB (TNM 7-th Ed.) after tri-modal treatment, but again no information regarding PTV was reported (Peters et al. 2019; Durm et al. 2020).

The currently ongoing BRIDGE trial aims to evaluate the proportion of patients who did not progress and who achieved a mean lung dose < 20 Gy and/or a lung V20 < 35% after combined immune- and chemotherapy prior to TRT (Mario Negri Institute for Pharmacological Research 2022). In our study, V20 ≥ 30 was found to be associated with LRRFS and OS exclusively in patients treated without ICI. We observed that V20 ≥ 30 was less common amongst patients treated with VMAT and/or PTV < 700 cc. Another possible explanation of these findings may be more strict attention to pulmonary constrains (V20 and MLD) after presentation of PACIFIC data.

In 2018, Driessen et al. published an analysis, in which they stated that CRT was more frequently applied in patients aged 65 to 74 years compared to patients over 75 years. Furthermore, differences in survival mostly disappeared after stratification by treatment (Driessen et al. 2018). In our analysis, all patients completed cCRT and there was a significant advantage in OS for patients < 65 years in the entire cohort, as well as a trend for better survival in patients < 65 years that did not receive ICI. However, no impact of age on PFS could be documented. Our findings regarding 20 + PY were similar.

Several studies have described the influence of SUV_max_ on outcome in patients with advanced NSCLC. In their 2011 analysis, Huang et al. demonstrated that changes in PET parameters, such as SUV and MTV, correlated significantly with outcome in patients with advanced NSCLC (Huang et al. 2011). Similarly, Wei et al. observed a negative impact of SUV_max_ on PFS (*p* < 0.05), as well as OS (*p* = 0.005) (Wei et al. 2022). Ercelep et al. defined 12 as the ideal cut-off value of pretreatment SUV_max_ for inoperable stage III NSCLC patients. They observed complete response in 60% of patients with SUV_max_ < 12 vs. 19% with SUV_max_ ≥ 12 (*p* = 0.002). Also, SUV_max_ ≥ 12 was a prognosticator for OS (*p* = 0.087) (Ercelep et al. 2019). In our analysis, SUV_max_ ≥ 13.75 was derived by median dichotomization. While we observed no significant impact on patient outcome in the univariate analysis of the entire cohort, it was highly significant for outcome amongst non-ICI patients. These findings were confirmed in the multivariate analysis. In addition, other PET parameters have been shown to be significantly associated with patient outcome (Eze et al. 2021). More recently, our group demonstrated that residual metabolic tumor volume (rMTV) was a strong predictor of outcome in stage III NSCLC patients undergoing CRT without ICI (Unterrainer et al. 2021).

In conclusion, all possible strategies for potential reduction of PTV should be implemented, including functional (PET/CT) and invasive (EBUS, EUS, mediastinoscopy) diagnostics, analysis/reduction of tumor motion and strict image guidance during radiation treatment. Intensified induction regimes including chemoimmunotherapy could therefore further improve this new tri-modal approach by ensuring a reduction in the GTV and consequently PTV.

## Conclusion

The present study found that patients with inoperable stage III NSCLC treated after 2018 demonstrated improved survival regardless of ICI use. However, implementation of ICI resulted in further improvement of patient outcome. PTV ≥ 700 cc and ICI were the only prognosticators of LRRFS, PFS and OS in the entire cohort. Further optimization strategies should consider both parameters and their interaction as most relevant for long-term patient outcome.
